# Recombineering in *Corynebacterium glutamicum* combined with optical nanosensors: a general strategy for fast producer strain generation

**DOI:** 10.1093/nar/gkt312

**Published:** 2013-04-28

**Authors:** Stephan Binder, Solvej Siedler, Jan Marienhagen, Michael Bott, Lothar Eggeling

**Affiliations:** Institut für Bio- und Geowissenschaften, IBG-1: Biotechnologie, Forschungszentrum Jülich GmbH, D-52425 Jülich, Germany

## Abstract

Recombineering in bacteria is a powerful technique for genome reconstruction, but until now, it was not generally applicable for development of small-molecule producers because of the inconspicuous phenotype of most compounds of biotechnological relevance. Here, we establish recombineering for *Corynebacterium glutamicum* using RecT of prophage Rac and combine this with our recently developed nanosensor technology, which enables the detection and isolation of productive mutants at the single-cell level via fluorescence-activated cell sorting (FACS). We call this new technology RecFACS, which we use for genomic site-directed saturation mutagenesis without relying on pre-constructed libraries to directly isolate l-lysine-producing cells. A mixture of 19 different oligonucleotides was used targeting codon 81 in *murE* of the wild-type, at a locus where one single mutation is known to cause l-lysine production. Using RecFACS, productive mutants were screened and isolated. Sequencing revealed 12 different amino acid exchanges in the targeted *murE* codon, which caused different l-lysine production titers. Apart from introducing a rapid genome construction technology for *C. glutamicum*, the present work demonstrates that RecFACS is suitable to simply create producers as well as genetic diversity in one single step, thus establishing a new general concept in synthetic biology.

## INTRODUCTION

For the development of microbial producer strains, fast methodologies are required for introducing genomic mutations. They inevitably have to go beyond serial and tedious introduction of single-DNA constructs into cells and identification of the desired mutations. Recombineering offers such a methodology and enables the rapid introduction of a single mutation into the genome (1–3), or even numbers of mutations in one experiment, such as exemplified in a multiplex automated engineering approach for *Escherichia coli* (4). However, to make full use of recombineering in producer strain generation, also a rapid detection of those recombinants that cause improved synthesis of the desired product is required. In case the product is colored, a visual inspection of colonies is possible (5), but even in this case, the library size that can be screened is limited. Even worse, most small molecules of interest are not colored. The problem of ultrahigh-throughput detection and isolation of productive recombinants has recently been solved by the development of optical nanosensors based on transcription factors. They allow the detection of intracellularly synthesized small molecules at the single-cell level (6–8), and, together with fluorescence-activated cell sorting (FACS), they enable the ultrahigh-throughput screening of large libraries. We have developed an l-lysine sensor for *Corynebacterium glutamicum,* which transmits the cytosolic l-lysine concentration of a single cell into a graded optical output. Using this sensor, we isolated new l-lysine producers via FACS from a library of 10^7^ randomly mutagenized wild-type cells and identified novel relevant mutations causing increased l-lysine synthesis by whole-genome sequencing (6).

The bacterium *C. glutamicum* is of particular interest, as it is one of the most important metabolite producers used in industry (9). Mutants of *Corynebacterium* are used to produce ∼2 500 000 tons l-glutamate, 1 800 000 tons l-lysine and 15 000 tons nucleotides annually. Driven by the need for the rapid introduction of chromosomal mutations, we asked whether recombineering in *C. glutamicum* would be possible. Recombineering takes advantage of phage-encoded recombination proteins and has greatly improved genetic manipulation in *E. coli* in a simple and efficient manner (2). The phage λ recombination proteins Exo, Beta and Gam—as well as RecE and RecT encoded by the *E. coli* Rac prophage—promote homologous recombination at a high frequency requiring only short stretches of homologous DNA sequences (10,11). The Exo and RecE proteins are double-stranded DNA (dsDNA)-dependent 5′−3′ exonucleases (12), Beta and RecT are single-stranded DNA (ssDNA) annealing proteins (SSAPs). They are capable of annealing homologous DNA (13) to perform strand exchange and strand invasion and are, therefore, also called recombinases. Exonuclease and recombinase together facilitate homologous exchange of dsDNA substrates (14). Synthetic ssDNA substrates (oligos) recombine efficiently to generate point mutations, deletions and insertions, and they require only recombinase activity (15). Beta and RecT, respectively, their homologs, facilitate recombineering in *Salmonella enterica*, *Lactobacillus, Bacillus subtilis* and other bacteria (1,16–19). However, they work less efficiently in *Mycobacterium smegmatis* (20). More recently, the RecE and RecT homologs, Gp60 and Gp61, from mycobacteriophage Che9c were demonstrated to encode recombination proteins and used to establish allelic exchange in *M. smegmatis* and *Mycobacterium tuberculosis* (21). Also the more distantly related two recombineering proteins of mycobacteriophage Halo were used for gene replacement and introduction of point mutations into mycobacterial genomes (3).

Recombineering as demonstrated for a few species stimulated us to ask whether this technology can be established for *C. glutamicum*. Our metabolite sensors are of extraordinary profit in this context. The reason is that recombineering has been almost exclusively used to date in plate assays for selectable and visible phenotypes, such as resistance to a compound or colony color. Our metabolite sensor technology is based on the expression of *eyfp* in response to elevated small-molecule concentrations within cells. This enables FACS screening of single cells, which in conjunction with recombineering establishes a new general concept for genome engineering in synthetic biology.

## MATERIALS AND METHODS

### Bacterial strains

For recombinant DNA work, *E. coli* DH5α was used. The *C. glutamicum* strain used was the type strain ATCC13032 and its derivatives, which were grown at 30°C as previously described (9). The *C. glutamicum* test strain used to establish recombineering in *C. glutamicum* was derived from DM1728. The test strain contained at nucleotide 1 404 651 (Accession number: NC_006958) a defective kanamycin resistance gene with an additional cytosine inserted at position 234 introducing a frameshift mutation. This strain was constructed by plasmid-based homologous recombination using pK19mobsacB-Kan(−) containing the defective gene, as well as genomic sequences adjacent to the insertion site (22). The resulting strain DM1728-Kan(−) served as a tester strain for the development of recombineering, whereas RecT-aided producer strain development was based on *C. glutamicum* ATCC13032. All recombinant strains generated were transformed by electroporation.

### Plasmid constructions and oligonucleotides

The genes for the recombinases Gp43, Gp61 and RCau were synthesized (LifeTechnologies GmbH, Darmstadt, Germany) and cloned into pCLTON2, which confers spectinomycin resistance to *C. glutamicum*. Fragments were generated with BglII and EcoRI, made blunt and cloned into the SmaI site of pCLTON2 to generate pCLTON2-gp43, pCLTON2-gp61 and pCLTON2-rCau, respectively. To construct pCLTON2-bet, bet was amplified from plasmid pRSFRedkan (23) using primer pair bet-F/bet-R and cloned blunt-end into pCLTON2. For the construction of pCLTON2-recT, the gene was amplified from the genome of *E. coli* MG1655 using primer pair recT-F/recT-R and cloned blunt-end into pCLTON2. Plasmid pEKEx3-recT was constructed using BglII-RBS-recT-F and EcoRI-recT-R for amplification and cloned using BglII and EcoRI into pEKEx3 resulting in pEKEx3-recT. Primers used for cloning and oligos used for recombineering were purchased from Eurofins MWG Operon (Ebersberg, Germany). They were salt free, without a 5′-phosphate, and they are listed in Supplementary Tables S1 and S2, respectively.

### Preparation of cells for recombineering

Strains of *C. glutamicum* DM1728-Kan(−) carrying pCLTON2 or pEKEx3 derivatives encoding recombinases were inoculated from a fresh BHIS-Spec100 Petri dish (9) into 50 ml of BHIS-Spec100 and grown for 16 h at 30°C and 120 rpm. Ten milliliters of this pre-culture was used to inoculate 500 ml of BHIS-Spec100. In addition, cultures containing pEKEx3 derivatives were supplemented with 0.5 mM IPTG, and cultures containing pCLTON2 derivatives received 250 ng/ml of anhydrotetracyclin, which served for induction of the recombinases. Five hours later, cells were harvested and made electrocompetent. They were chilled on ice for 20 min and then harvested at 4000 rpm and 4°C for 20 min, washed twice in 50 ml of TG-buffer (1 mM Tris–HCl, pH 8, and 10% glycerol) and twice in 50 ml of glycerol 10%. The competent cells were then re-suspended in 1-ml 10% glycerol and 150 µl of aliquots stored at −70°C before use. Use of fresh unfrozen cells might result in higher recombineering frequencies (41).

To prepare ATCC13032 pSenLys pEKEx3-recT for recombineering, media additionally contained kanamycin to select for pSenLys (plates 15 µg ml^−^^1^ and liquid media 25 µg ml^−^^1^).

### Recombineering assay for repair of defective KanR

Electrocompetent cells of DM1728-KanR(−) carrying the plasmid with the recombinase to be assayed were thawed on ice and mixed with 0.1–100 µg of ssDNA oligos and transferred into 4°C pre-cooled electroporation cuvettes. Electroporation was performed at 25 µF, 200 Ω and 2.5 kV. Subsequently cells were immediately transferred into 4 ml of pre-warmed BHIS medium containing 100 µg/ml of spectinomycin and heat shocked for 6 min at 46°C in a water-bath. They were allowed to regenerate and segregate for up to 5 h at 30°C and 170 rpm. Cells were plated on BHIS-Spec100-Kan15 and incubated at 30°C for 2 days for cfu determination. As a negative control, an oligonucleotide with no sequence similarity to the *C. glutamicum* genome was added to one aliquot of electrocompetent cells. In addition, one aliquot was transformed with 100 ng of pJC1 conferring kanamycin resistance to determine competence and transformation efficiency.

### Recombineering assay for generating l-lysine producers

Electrocompetent cells of *C. glutamicum* ATCC13032 pSenLys pEKEx3-recT were prepared as described earlier in the text. For the generation of recombinant strains carrying the *lysC-*T311I mutation, 20 µg of the lysC-60-EcoRV*-oligo was used. For *in vivo* site-directed saturation mutagenesis of *murE*-81, a mixture of 20 oligos (1 µg of each 100 mer and 20 µg in total) was used for electroporation. After electroporation and regeneration for 5 h, 100 µl of the cell suspension was centrifuged (5 min, 4000 rpm, 4°C) washed once with CGXII, re-suspended in 800 µl of CGXII-Spec100-Kan25 and transferred into a flowerplate, FP, (m2p-labs GmbH, Baesweiler, Germany) for further cultivation for 48 h at 30°C, 900 rpm and a throw of 3 mm.

### FACS analysis and two-step HT-screening

For FACS analysis, all samples were diluted to an optical density <0.1 and immediately analyzed by a FACS ARIA II high-speed cell sorter (BD Biosciences, Franklin Lakes, NJ, USA) using excitation lines at 488 and 633 nm and detecting fluorescence at 530 ± 15 and 660 ± 10 nm at a sample pressure of 70 psi and a processing rate of ∼10 000 cells per second. Data were analyzed using the BD DIVA 6.1.3 software. As sheath fluid sterile-filtered phosphate-buffered saline was used. Electronic gating was set to exclude non-bacterial particles on the basis of forward versus side scatter area. For further gating the l-lysine-producing strain *C. glutamicum* DM1728, pSenLys pEKEx-recT served as a positive control, and the wild-type with the two plasmids was used as a negative control. These strains were electroporated and cultivated in parallel to the actual sample.

Before FACS selection, 8 µl of the cells cultivated for 48 h were inoculated into fresh 800 µl of CGXII-Spec100-Kan25 and grown for 2–5 h as described earlier in the text. The two-step screening routine consisted of an enrichment step where 10 000 fluorescent cells were spotted into 800 µl of CGXII-Spec100-Kan15 followed by 48 h of cultivation in FPs at 30°C and 900 rpm. Eight microliters of this culture served to inoculate 800 µl of fresh CGXII-Spec100-Kan25, which was grown for 2–5 h. This was followed by FACS selection and spotting of cells onto BHIS-Spec100-Kan15 plates. After incubation for 2 days at 30°C, clones were further analyzed in terms of fluorescence and product formation.

### HT cultivation and amino acid analysis

HT cultivation was done in 48-well FPs at 30°C, 900 rpm and a throw of 3 mm. The specific geometry of the FPs ensures a high mass-transfer performance and can be used together with the microcultivation system BioLector (24), allowing online monitoring of growth and fluorescence. For offline cultivations, FPs were cultivated on a Microtron high-capacity microplate incubator operating at a shaker speed of 900 rpm, throw of 3 mm (Infors AG, CH-4103 Bottmingen, Switzerland) for 48 h until all cultures reached the stationary phase. Offline fluorescence determinations were done by mixing 5 µl of the culture with 195 µl of H_2_O and using a Tecan microplate reader. The cultures were excited at 500 nm and emission quantified at 530 nm.

Amino acids were quantified as their *o*-phthaldialdehyde derivatives via high-pressure liquid chromatography using an uHPLC 1290 Infinity system (Agilent, Santa Clara, CA, USA) equipped with a Zorbax Eclipse AAA C18 3.5 μm 4.6 × 75 mm and a fluorescence detector as described previously (6). Cultivations were done at least twice and gave comparable results.

## RESULTS

### Selection of recombinases

**Figure 1.**
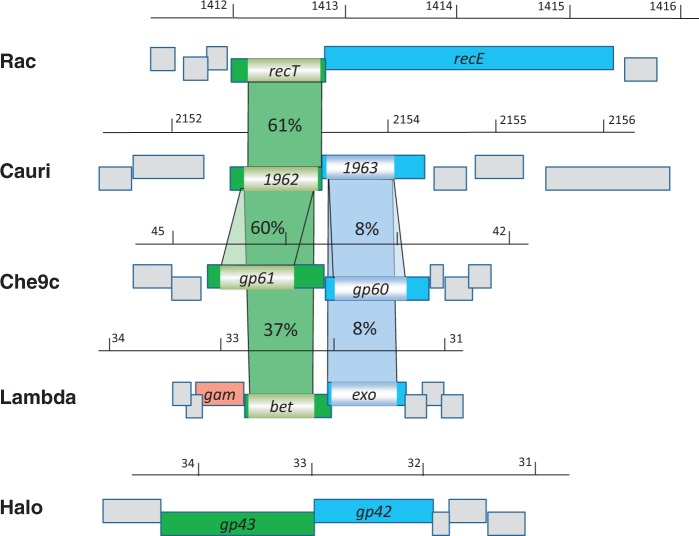
Comparison of the *C. aurimucosum* recombination proteins. The cauri_1962 gene product contains a RecT domain, as does gp61 and Bet, with highest homology of cauri_1962 to the prophage Rac protein and gp61. Halo gp43 is not related to these ssDNA-binding proteins. The putative exonuclease cauri_1963 belongs to the YqaJ family of exonucleases. cauri_1963 is only distantly related to the gp60 protein and Exo, and it is unrelated to RecE and gp42. The genome of *C. aurimucosum* does not contain a λ Gam homolog (red).

Single-stranded DNA (ssDNA) annealing proteins (SSAPs) play critical roles in recombination-dependent DNA replication in any organism, with specific subclasses typical for bacteriophages and prophages (25,26). Using RecT of the Rac prophage as a query sequence, we screened in a Basic Local Alignment Search Tool search for homologs within 25 genomes of *Corynebacterium* species. The sole homolog identified was cauri_1962 of *Corynebacterium aurimucosum* (Figure 1), which has 61% similarity with RecT. Adjacent and co-transcribed to cauri_1962 is a gene encoding a viral exonuclease domain. This protein has no significant similarity to the exconuclease RecE. For our initial studies to assay on the functionality of recombinases in *C. glutamicum,* we focused on RecT and cauri_1962. In addition, we chose the well-studied λ red gene *bet*, as well as the SSAPs of the mycobacteriophage Che9c and Halo. The reason for this is that both *Mycobacterium* and *Corynebacterium* belong to the order *Corynebacteriales*, and genes of *M. tuberculosis* show functionality in *C. glutamicum* (27,28). Cauri_1962 is also closely related to Gp61 of Che9c and Bet, but it has no similarity to Gp43 of Halo. Cauri_1963, the putative exonuclease, has a weak similarity to Gp60 of Che9c and Exo of λ, but no similarity to RecE or the Halo protein Gp42. Thus, the exonucleases seem to be more species specific, whereas the recombinases tend to be conserved. This agrees with functional studies on recombinase/exonuclease pairs from diverse bacteria in *E. coli*: selected pairs displayed good recombination activity with ssDNA, but they were less efficient with dsDNA, the latter requiring both activities (29).

### Functionality of recombinases

We used the expression vector pCLTON2 to clone the five selected recombinases, which is based on the tetracycline inducible Tet repressor system derived from *E. coli* (30). The five constructs pCLTON2-bet, pCLTON2-recT, pCLTON2-gp43, pCLTON2-gp61 and pCLTON2-rCau — where rCau stands for the recombinase cauri_1962 —were introduced into *C. glutamicum* DM1728 Kan(−). This latter strain was the tester strain. It contained a defective kanamycin resistance gene integrated into its genome. It was constructed by using pK19mobsacB-Kan(−) encoding the Kan(−) gene with an additional cytosine inserted at position 234 introducing a frameshift and ultimately a truncated non-functional protein. Using this non-replicative vector, the defective Kan gene was placed in a non-coding region of the chromosome by two rounds of homologous recombination. This tester strain was transformed with the pCLTON2 derivatives, and the resulting strains were used in recombination assays.

In one early experiment, heterologous expression of the recombinase genes was induced with 250 ng ml^−^^1^ of anhydrotetracyclin. After 4 h of induction, cells were made electrocompetent and frozen. Electroporation was done with 1 µg of oligo Kan50*, which is a 50mer with the correct sequence part of KanR and homology to the leading strand. Cells were plated after regeneration for 5 h on BHIS-Kan15-Spec100. With pCLTON2-recT ∼12 500 and with pCLTON2-rCau 2500 KanR colonies per transformation assay were obtained (Table 1). This shows that RecT is functional in *C. glutamicum,* and rCau from *C. aurimucosum* encodes a functional protein that is also active in *C. glutamicum*. A weak activity was also obtained with gp61 from *M. smegmatis*. In controls where no oligo was added, at best 57 cfu were observed on BHI-Kan15-Spec100 plates. Each transformation assay was done with ∼10^9^ cells surviving electroporation. Such assays yielded 2.2 × 10^5^ to 5.0 × 10^5^ Kan^R^ cfu when transformed with the replicative plasmid pJC1 (Table 1), illustrating that the competence for uptake of DNA after expression of the recombinases is comparable with standard transformation conditions without protein expression (31). When the oligo-specific number of transformants was put in relation to that derived by the replicative plasmid, the recombinase RecT yielded 4.7 chromosomal recombinants per 100 cells capable of taking up plasmid. In these experiments, ∼40 000 oligo molecules per cell or 300 plasmid molecules, respectively, were present in the electroporation assay.

**Table 1.** Comparison of recombinase efficiencies

Vector	Km^R^ per 10^9^ viable cells		
	+oligo^a^	−oligo	+plasmid
pCLTON2-bet	8	0	2.2 × 10^5^
pCLTON2-recT	1.3 × 10^4^	31	5.0 × 10^5^
pCLTON2-gp43	9.7 × 10^1^	57	4.3 × 10^5^
pCLTON2-gp61	3.1 × 10^2^	1	3.4 × 10^5^
pCLTON2-rCau	2.5 × 10^3^	7	2.9 × 10^5^

^a^As oligo 1 µg the 50mer Kan* was used, and as plasmid 1 µg of the replicative vector pJC1. Values indicated are the average of three or more experiments.

### Optimization of recombineering

**Figure 2.**
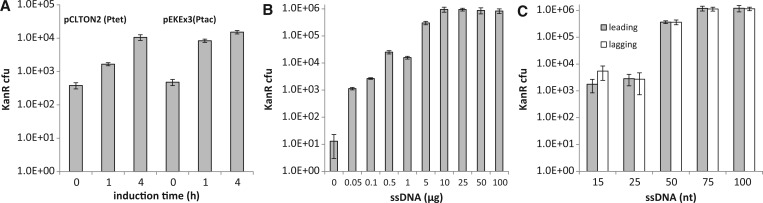
Optimization of recombineering efficiency. (**A**) Recombination efficiency of recT induced for 0, 1 and 4 h using either pCLTON2-recT and 0.25 mg/l of anhydrotetracyclin or pEKEx3-rect and 0.24 mg/l of IPTG. (**B**) Recombination efficiency depended on the amount of oligonucleotide added. (**C**) Recombination efficiency depended on length and strand homology. Data are the means of three experiments, with the standard deviation as indicated by the error bars.

As RecT was the most efficient recombinase in *C. glutamicum*, we next varied the induction time and used pEKEx3 as another vector background for recombinase expression (30). Cells of the tester strain *C. glutamicum* DM1728 Kan(−) carrying either pCLTON2-recT or pEKEx3-recT were induced for 0, 1 and 4 h. Transformation with 1 µg of the healing oligo Kan50* and regeneration was performed as described before. There was a clear increase in the number of recombinants with increasing induction time (Figure 2A). The highest numbers of recombinants were obtained with pEKEx3-recT. This is probably because of the known stronger expression of target genes in pEKEx3 compared with pCLTON2 (30). In all subsequent experiments, we, therefore, used pEKEx3-recT with the recombinase expression induced for 4 h and cell regeneration and segregation for 5 h.

With *E. coli*, concentrations of 0.2 µg of oligos are routinely used in recombineering assays (10), although in special applications, such as multiplex automated genome engineering, concentrations exceeding 20 µg are recommended (4). We assayed various oligonucleotide concentrations to optimize recombineering efficiency in *C. glutamicum* (Figure 2B). There was a strong increase in the number of recombinants with increasing oligo concentration, with as many as 9.5 × 10^5^ recombinants of 1 × 10^9^ cells surviving electroporation when using 10 µg of Kan*. This means that 1 of ∼1.000 cells is also recombinant. Higher concentrations did not improve the recombineering efficiency, but rather led to a slight decrease in the number of recombinants.

For a given target, there are two complementary ssDNA oligos, either one of which can be used for recombineering. The corresponding oligonucleotide that is complementary to the template strand for discontinuous DNA synthesis (i.e. the lagging strand) recombines ∼15- to 20-fold more efficiently in *E. coli* than the oligonucleotide complementary to the leading strand because of activity of the recombinase Beta (10,16). As further parameters for recombineering efficiency in *C. glutamicum*, we, therefore, assayed both complementary oligonucleotides, and we varied the lengths of the oligos. In these experiments, equimolar concentrations of 32.5 µmol were used, which correspond to 10 µg for the 100mer. As can be seen in Figure 2C, the use of a 75mer yields >10^6^ recombinants per assay, whereas a further increase in the length of the oligo did not increase recombination frequency. This largely agrees with the situation in *E. coli* where the highest level of recombinants is generated with a 60mer (10). Comparison of the two complementary oligo pairs yielded a slightly increased number of recombinants in *C. glutamicum* with oligos annealing to the lagging strand (Figure 2C). Thus, there is a strand bias, but this is less pronounced than in *E. coli*. With phage Che9c gp61-mediated recombination in *M. tuberculosis*, there is an even stronger strand bias and an oligonucleotide targeting lagging strand DNA synthesis can recombine >10 000-fold more efficiently than its complementary oligonucleotide (20). Thus, for ssDNA recombineering in *C. glutamicum*, there is no need to differentiate between the two complementary ssDNA oligos possible, and with the oligo length of choice, ∼10^6^ recombinants are obtained per assay.

We also performed experiments to reduce a possible mismatch repair during recombination, and we used C·C mismatches or mismatches at consecutive wobble positions nearby to the correcting base making silent mutations (Supplementary Figure S1). An up to 1.3-fold increase in recombineering frequency was obtained, demonstrating the importance of sequence context. This is rather low compared with the comparable experiments on *E. coli,* and it could indicate a difference in the mismatch repair system between these organisms, as indicated by comparisons of the repertoire of DNA-repairing enzymes in *Corynebacterium* species (32). However, this aspect warrants further studies.

### Recombineering and direct producer isolation by product sensing

Although selection for antibiotic resistance is useful to establish recombineering, it is of limited use for producer strain development. To overcome this problem, screening methods for non-selectable recombinants, such as hybridization of colonies, and other techniques were established (16). We developed optical sensors that respond to increased product formation in single cells by emitting fluorescence (6,33). These sensors provide a direct signal when a ‘productive mutation’ is introduced. It enables the selection of productive mutants in ultrahigh-throughput screens using FACS.

**Figure 3.**
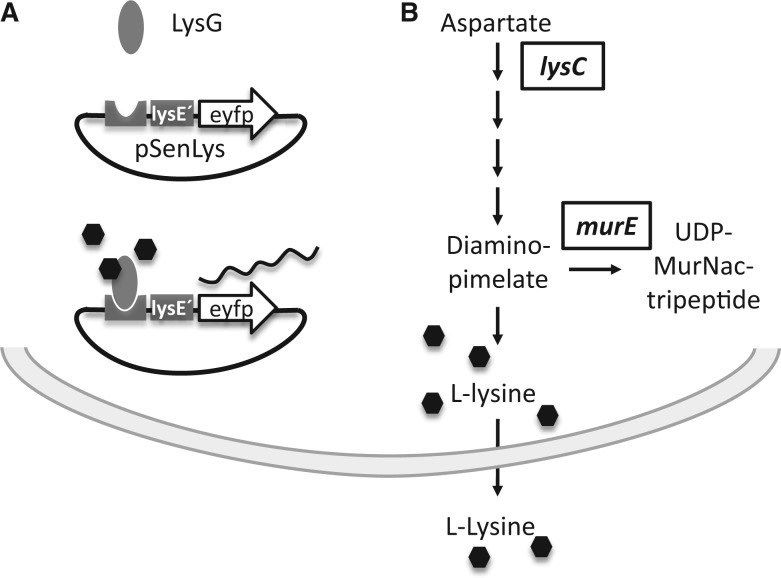
(**A**) Principle of l-lysine sensor pSenLys. The transcriptional regulator LysG senses l-lysine (34) to naturally drive transcription of its target gene *lysE*. When this is fused to *eyfp*, cells exhibit fluorescence at an increased cytosolic l-lysine concentration. (**B**) *lysC* and *murE* are two targets known in *C. glutamicum*, which on specific mutations provoke an excess l-lysine synthesis.

One metabolite sensor we have developed is pSenLys. It is based on the transcriptional regulator LysG of *C. glutamicum* (Figure 3), which recognizes increased l-lysine concentrations in the cytosol to drive transcription of its target gene *lysE* (34). The fusion of *lysE* with *eyfp* results in cells emitting increased fluorescence at elevated l-lysine concentrations (6). To assay for the use of this metabolite sensor and FACS screening of recombinants, we chose *lysC* as a recombineering target in the chromosome of *C. glutamicum*. *lysC* encodes the aspartate kinase, which controls the entry of l-aspartate into the l-lysine synthesis pathway (Figure 3). When the codon 311 of *lysC*, which is ACC, is changed to ATC, a threonine is replaced by isoleucine in the protein sequence. As a consequence, the kinase is no longer feedback inhibited by l-lysine, and the wild-type is converted into an l-lysine producer (35).

**Figure 4.**
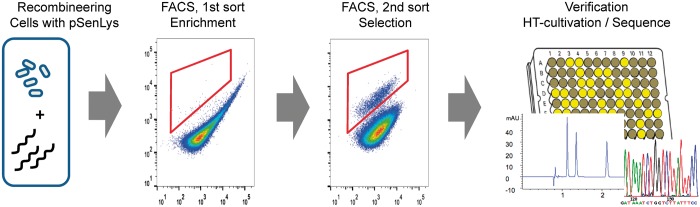
Principle of RecFACS as demonstrated for introduction of the productive *lysC*-T311I mutation. Recombineering was done with cells containing the metabolite sensor pSenLys. After recombineering, cells were grown in liquid culture on minimal medium and subjected to a first FACS sort to enrich positive clones in liquid culture. The enrichment cultures were used for the second FACS sort, where cells were spotted on Petri dishes and subsequently cultivated in a microtiter plate to follow growth and fluorescence. l-lysine was quantified in culture supernatants, and the correct genomic integration was finally verified by sequencing.

Cells of *C. glutamicum* pEKEx3-recT pSenLys were induced for expression of RecT and electroporated with 10 µg of the oligo lysC-60-EcoRV*. This 60mer corresponds to the non-coding strand and carries in the middle the sequence GTGAAG**A**T*GAT****A****TC*GG, with the nucleotides in bold being exchanged. The underlined codon introduces *lysC*-T311I, and the nucleotides in italics an adjacent EcoRV site. After regeneration and segregation, cells were grown in minimal medium CGXII-glucose, and cells with increased fluorescence were enriched via FACS. The gate for cell selection was chosen according to the fluorescence of the l-lysine producer SBK052 carrying pSenLys (Figure 4). Gating was done with a known producer and a non-producer (see ‘Materials and Methods’ section). The enrichment culture derived from the recombination assay was then analyzed again (second sort) with 2.4% of the cells exhibiting the increased fluorescence as expected for an l-lysine producer. This number was 0.05% for the negative control, which received water instead of the oligo. Cells with increased fluorescence of the second sort were spotted onto Petri dishes, and from 12 clones, the *lysC* target was amplified using polymerase chain reaction. The diagnostic restriction analysis revealed that the EvoRV restriction site was present in five clones resulting from the recombineering assay, whereas this was not the case for any clone of the negative control.

### One-step diversity generation and producer selection

To go even further with respect to the possibilities offered by the single-cell metabolite sensor and high-frequency recombineering, we aimed to generate genetic diversity at a given locus across a population in one single experiment. A related attempt has been successfully demonstrated for *E. coli* and *lacZ* as a target via multiplex automated genome engineering (4). Combining diversity generation by recombineering with our sensor technology should allow the direct selection of diverse productive cells. The target gene chosen for this experiment was *murE* (Figure 3). In previous work, we identified this gene by whole-genome sequencing as an attractive target to engineer l-lysine synthesis, and found that the specific mutation *murE*-G81E results in increased l-lysine titers (6).

**Figure 5.**
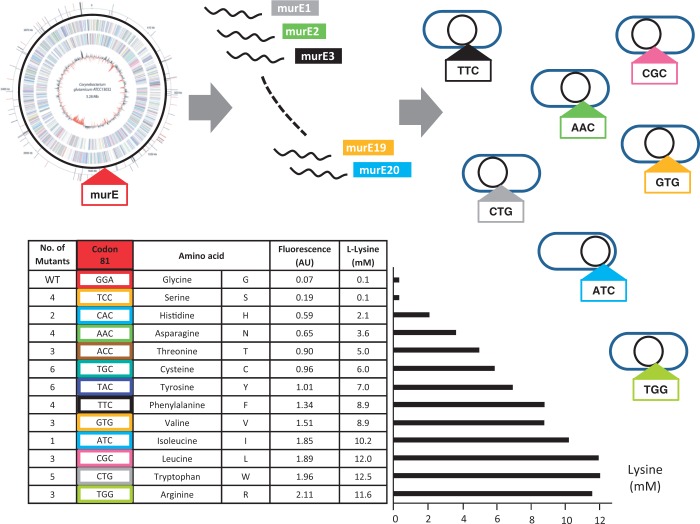
RecFACS to create producers with genetic diversity at codon 81 of *murE* in the genome of *C. glutamicum* wild-type. A mixture of oligonucleotides murE1 to murE20 was used for ssDNA recombineering, and recombinants with productive mutations were selected via FACS. Thirteen of the 20 possible recombinants were obtained (table), exhibiting l-lysine formation to different degrees (black bar).

Twenty different 100mer oligos were designed, each with two to three nucleotides exchanged in the middle of the sequence to introduce the respective codons for any of the 19 amino acids in position 81 of *murE*, except the original codon and plus one oligo with stop codon (Supplementary Table S2). A mixture of these oligos, 1 µg each, was added to recombineering competent *C. glutamicum* pEKEx3-recT pSenLys (Figure 5). Regenerated cells were inoculated as described earlier in the text on minimal medium, and cells were screened via FACS without an enrichment step. We set a sorting gate based on positive and negative controls, which would collect all 10 000 cells analyzed from DM1728 pEKEx3-recT pSenLys but only eight cells from WT pSenLys. Using this gate, 10^5^ cells of the recombineering assay were analyzed, and 220 positive cells were spotted onto a Petri dish. Using tooth picks, 132 clones were inoculated into 0.8 ml of CGXII-glucose in flower plate wells. In total, 126 cultures grew, of which 53 exhibited increased fluorescence. The oligos were designed in such a way that a chromosomal PvuII restriction site would disappear on successful recombineering. A diagnostic restriction analysis revealed that this was the case in 21 clones, which were subsequently sequenced. One clone was obtained in which G in position 81 of MurE was replaced by V, and two or more clones where a C, F, L, N, S, W or Y was present in this position. The experiment was repeated starting from new recombineering proficient cells and yielded a comparable result.

All data and analyses of individual clones are given in Supplementary Table S3. The combined data are given in the table included in Figure 5. In total, 44 recombinants were derived, a broad range of substitutions were obtained and G81 was replaced by 12 different amino acids. The mutations were ranked in the table according to the fluorescence of the respective cultures, and this largely agrees as expected with l-lysine accumulation. With the exception of the G81S mutation, all mutations produced l-lysine. Furthermore, the production of individual clones carrying the same mutation is largely consistent (Supplementary Table S3). Thus, RecFACS, the combined use of metabolite sensors in conjunction with recombineering, allows in one single step the selection of productive mutants exhibiting a range of genetic diversity at a defined genomic locus.

## DISCUSSION

Recombineering with ssDNA is easy and rapid to perform requiring just the presence of an SSAP. It has already been realized for different bacteria. Gammaproteobacteria clearly represent the largest group for which recombineering has been demonstrated (1,2), and its application is best developed for *E. coli* (4,36). Recombineering has additionally been demonstrated for a few other bacteria including *Bacilli* and *Mycobacterium* species (3,18,19). In the present work, we assayed five different SSAPs for functionality in *C. glutamicum*, among them cauri_1962. This is present in the genome of *C. aurimucosum* at a locus where phage-related proteins occur (37), and our work demonstrates that the protein encodes a functional recombinase.

The highest recombineering activity in *C. glutamicum* is obtained with RecT enabling frequencies exceeding 10^6^ recombinants per assay, which contained 10^9^ viable cells after electroporation. This is only about one order of magnitude away from that obtained with Bet in *E. coli* with a proper genetic background like with a decreased activity of the methyl-directed mismatch repair system (10). Apart from the possible use of such mutations in *C. glutamicum*, it is evident from the variation of vector use and induction time that there is still room for further optimization of the recombineering system in *C. glutamicum*. Significant activities were also obtained with gp43 and gp61. In these latter cases, the formation of the corresponding SSAPs might be limiting because the codon adaptation index is <0.29 for both genes, suggesting weak protein formation.

Although selectable phenotypes, e.g. drug resistance or auxotrophy, are useful for the development of recombineering, this is less so the case if the production quality for a small molecule is to be improved. The reason is 2-fold: first, small molecules usually have an inconspicuous phenotype, and second, plate assays for phenotypic selections are limited with respect to the number of clones that can be analyzed. To overcome limitations in the absence of a direct selectable phenotype, a two-step protocol for *E. coli* can be used to modify a region of interest, where in the first step the target region is replaced by *cat-sacB* as a dual selection cassette (16). A recent technique developed for *E. coli* is the simultaneous use of two oligos with one of them introducing a selectable marker within 500 kb of the second target (36). As the frequency of co-selection of the second target is up to 3-fold higher than without selection, the numbers of clones to be characterized by subsequent PCR analysis are reduced.

Our previously developed sensor technology provides an effective screen for single cells producing small molecules, such as amino acids (6). In contrast to plate assays, it is an ultrahigh-throughput technology. Its use together with recombineering — a procedure we call RecFACS — opens up a number of exciting possibilities to engineer genomes with the direct selection of producers. As the first example, we demonstrated this for the nucleotide exchange C → T at position 932 of the aspartate kinase of *C. glutamicum* resulting in LysC-T311I. The mutation reduces feedback control of the kinase, making the strain an l-lysine producer. As kinase mutations are known to control the enzyme activity to different degrees (38), these — or other mutations known to increase l-lysine productivity — can now easily be introduced in strains to assay for their consequences on l-lysine formation.

RecFACS enables the direct selection of productive mutations. Even better, it allows the introduction of genetic diversity during producer creation. This is an additional quality of RecFACS, as we have demonstrated for *murE* when we performed target-specific random mutagenesis. In one single experiment, a range of substitutions of MurE-G81 was obtained. In addition, the correlation between fluorescence of the isolates and their l-lysine accumulation is given, as we expected from the increasing cytosolic steady-state concentrations of producers, which correlate with sensor responsiveness (6). A few clones of the same mutation were potential outliers. This can simply be counteracted by analyzing a larger number of clones.

Some of the 20 mutagenic oligos did not result in productive isolates by RecFACS, and there are various explanations for this. Among them is recombineering itself. The pool of 100mers we used differed over a sequence of up to four nucleotides, and it has been demonstrated that oligos with more homology to the target will be incorporated into the chromosome at a higher frequency than those with less homology (4). Moreover, although the DNA repair system of *C. glutamicum* is poorly defined (32), methyl-directed mismatch repair of *E. coli* is sensitive to sequence context, which can lead to an ∼100-fold variation in oligo recombination frequency (39). Another reason for the absence of some amino acid substitutions is that they are either non-productive or that they result in poor or even absent UDP-*N*-acetylmuramoyl-l-alanyl-d-glutamate:meso-diaminopimelate ligase activity. This particular activity is required for cell wall synthesis and is essential for *E. coli* (40). Enzymes with poor activity will result in poorer growth and might be outgrown before the FACS selection step of RecFACS.

In summary, extensive genome recombineering as achieved in *E. coli* is now accessible for *C. glutamicum*. Even more importantly, the potential of recombineering is significantly increased because of its alliance with metabolite sensors and establishment of RecFACS. Genetic diversity of productive mutants can be readily created. The technology can easily be extended and is expected to boost microbial strain development for small-molecule production in general.

## SUPPLEMENTARY DATA

Supplementary Data are available at NAR Online: Supplementary Tables 1–3 and Supplementary Figure 1.

## FUNDING

BMBF [0315589A] ‘Corynebacterium: Improving Flexibility and Fitness for Industrial Production’. Funding for open access charge: Research Centre Juelich.

*Conflict of interest statement.* None declared.

## Supplementary Material

Supplementary Data
