# Supplementary material for: Recombineering in Corynebacterium glutamicum combined with optical nanosensors: a general strategy for fast producer strain generation
Source: Nucleic Acids Res. 2013 Apr 28;41(12):6360–9. doi: 10.1093/nar/gkt312 (PMC3695502; doi:10.1093/nar/gkt312)
Supplement: Supplementary Data [file supp_41_12_6360__index.html]

Recombineering in Corynebacterium glutamicum combined with optical nanosensors: a general strategy for fast producer strain generation — Recombineering in Corynebacterium glutamicum combined with optical nanosensors: a general strategy for fast producer strain generation — Recombineering in Corynebacterium glutamicum combined with optical nanosensors: a general strategy for fast producer strain generation — Supplementary Data 

# Recombineering in *Corynebacterium glutamicum* combined with optical nanosensors: a general strategy for fast producer strain generation

## Supplementary Data

files

**Files in this Data Supplement:**

- Supplementary Data - docx file
