# Supplementary material for: Recombineering in Corynebacterium glutamicum combined with optical nanosensors: a general strategy for fast producer strain generation
Source: Nucleic Acids Res. 2013 Apr 28;41(12):6360–9. doi: 10.1093/nar/gkt312 (PMC3695502; doi:10.1093/nar/gkt312)
Supplement: Supplementary Data [file supp_gkt312_nar-00565-h-2013-File002.docx]

**Table S1:** List of primers used for plasmid constructions

| **Primer** | **Sequence** | **Construction of** |
| --- | --- | --- |
| Bet-F | AAGGAGATATAGATATGAGTACTGCACTCGCAAC | pCLTON2-bet |
| Bet-R | TCATGCTGCCACCTTCTGCTC |  |
| recT-F | AAGGAGATATAGATATGACTAAGCAACCACCAATC | pCLTON3-recT |
| recT-R | CGGTTATTCCTCTGAATTATCG |  |
| BglII-RBS-recT-F | GCAGATCTAAGGAGATATACATATGACTAAGCAACCACCAATCG | pEKEx3-recT |
| EcoRI-recT-R | GCGCGAATTCCAGGCTGAATTATTCCTC |  |

**Table S2:** List of oligonucleotides used

| **Lagging strand oligos** | |
| --- | --- |
| Kan100* | ATGCATCATCAGGAGTACGGATAAAATGCTTGATGGTCGGAAGAGGCATAAATTCCGTCAGCCAGTTTAGTCTGACCATCTCATCTGTAACATCATTGGC |
| Kan75* | AGTACGGATAAAATGCTTGATGGTCGGAAGAGGCATAAATTCCGTCAGCCAGTTTAGTCTGACCATCTCATCTGT |
| Kan50* | ATGCTTGATGGTCGGAAGAGGCATAAATTCCGTCAGCCAGTTTAGTCTGA |
| Kan25* | GGAAGAGGCATAAATTCCGTCAGCC |
| Kan15* | GAGGCATAAATTCCG |
| **Leading strand oligos** | |
| Kan100 | GCCAATGATGTTACAGATGAGATGGTCAGACTAAACTGGCTGACGGAATTTATGCCTCTTCCGACCATCAAGCATTTTATCCGTACTCCTGATGATGCAT |
| Kan75 | ACAGATGAGATGGTCAGACTAAACTGGCTGACGGAATTTATGCCTCTTCCGACCATCAAGCATTTTATCCGTACT |
| Kan50 | TCAGACTAAACTGGCTGACGGAATTTATGCCTCTTCCGACCATCAAGCAT |
| Kan25 | GGCTGACGGAATTTATGCCTCTTCC |
| Kan15 | CGGAATTTATGCCTC |
| **Oligos to assay for mismatch repair** | |
| Kan100 | GCCAATGATGTTACAGATGAGATGGTCAGACTAAACTGGCTGACGGAATTTATGCCTCTTCCGACCATCAAGCATTTTATCCGTACTCCTGATGATGCAT |
| Kan100-a48g | GCCAATGATGTTACAGATGAGATGGTCAGACTAAACTGGCTGACGGAGTTTATGCCTCTTCCGACCATCAAGCATTTTATCCGTACTCCTGATGATGCAT |
| Kan100-g45c | GCCAATGATGTTACAGATGAGATGGTCAGACTAAACTGGCTGACCGAATTTATGCCTCTTCCGACCATCAAGCATTTTATCCGTACTCCTGATGATGCAT |
| Kan100-g42c | GCCAATGATGTTACAGATGAGATGGTCAGACTAAACTGGCTCACGGAATTTATGCCTCTTCCGACCATCAAGCATTTTATCCGTACTCCTGATGATGCAT |
| Kan100-t51c | GCCAATGATGTTACAGATGAGATGGTCAGACTAAACTGGCTGACGGAATTCATGCCTCTTCCGACCATCAAGCATTTTATCCGTACTCCTGATGATGCAT |
| Kan100-t57c | GCCAATGATGTTACAGATGAGATGGTCAGACTAAACTGGCTGACGGAATTTATGCCCCTTCCGACCATCAAGCATTTTATCCGTACTCCTGATGATGCAT |
| Kan100-all | GCCAATGATGTTACAGATGAGATGGTCAGACTAAACTGGCTcACcGAgTTcATGCCcCTTCCGACCATCAAGCATTTTATCCGTACTCCTGATGATGCAT |
| Kan100* | ATGCATCATCAGGAGTACGGATAAAATGCTTGATGGTCGGAAGAGGCATAAATTCCGTCAGCCAGTTTAGTCTGACCATCTCATCTGTAACATCATTGGC |
| Kan100*-a48g | ATGCATCATCAGGAGTACGGATAAAATGCTTGATGGTCGGAAGAGGCATAAACTCCGTCAGCCAGTTTAGTCTGACCATCTCATCTGTAACATCATTGGC |
| Kan100*-g45c | ATGCATCATCAGGAGTACGGATAAAATGCTTGATGGTCGGAAGAGGCATAAATTCGGTCAGCCAGTTTAGTCTGACCATCTCATCTGTAACATCATTGGC |
| Kan100*-g42c | ATGCATCATCAGGAGTACGGATAAAATGCTTGATGGTCGGAAGAGGCATAAATTCCGTGAGCCAGTTTAGTCTGACCATCTCATCTGTAACATCATTGGC |
| Kan100*-t51c | ATGCATCATCAGGAGTACGGATAAAATGCTTGATGGTCGGAAGAGGCATGAATTCCGTCAGCCAGTTTAGTCTGACCATCTCATCTGTAACATCATTGGC |
| Kan100*-t57c | ATGCATCATCAGGAGTACGGATAAAATGCTTGATGGTCGGAAGGGGCATAAATTCCGTCAGCCAGTTTAGTCTGACCATCTCATCTGTAACATCATTGGC |
| Kan100*-all | ATGCATCATCAGGAGTACGGATAAAATGCTTGATGGTCGGAAGgGGCATgAAcTCgGTgAGCCAGTTTAGTCTGACCATCTCATCTGTAACATCATTGGC |
| **Oligo for engineering of *C. glutamicum* *lysC*** | |
| lysC_60_EcoRV* | CGGCGGCCGTCGGAACGAGGGCAGGTGAAGATGATATCGGTGGTGCCGTCTTCTACAGAA |
| **Oligos for engineering of *C. glutamicum* *murE*** | |
| G81amb | AACAACGATGACTGGGCGGGTCTCTCCTGCTTCGTTGAGCACCTCAAGTCAAGCTGCGTCAGTCAAAATGGCCACAGCTTTCGCAGCGTTATCCGTACCT |
| G81A | AACAACGATGACTGGGCGGGTCTCTCCTGCTTCGTTGAGCACCTCAAGTGCAGCGGCGTCAGTCAAAATGGCCACAGCTTTCGCAGCGTTATCCGTACCT |
| G81C | AACAACGATGACTGGGCGGGTCTCTCCTGCTTCGTTGAGCACCTCAAGGCAAGCTGCGTCAGTCAAAATGGCCACAGCTTTCGCAGCGTTATCCGTACCT |
| G81D | AACAACGATGACTGGGCGGGTCTCTCCTGCTTCGTTGAGCACCTCAAGGTCAGCGGCGTCAGTCAAAATGGCCACAGCTTTCGCAGCGTTATCCGTACCT |
| G81E | AACAACGATGACTGGGCGGGTCTCTCCTGCTTCGTTGAGCACCTCAAGTTCAGCGGCGTCAGTCAAAATGGCCACAGCTTTCGCAGCGTTATCCGTACCT |
| G81F | AACAACGATGACTGGGCGGGTCTCTCCTGCTTCGTTGAGCACCTCAAGGAAAGCTGCGTCAGTCAAAATGGCCACAGCTTTCGCAGCGTTATCCGTACCT |
| G81H | AACAACGATGACTGGGCGGGTCTCTCCTGCTTCGTTGAGCACCTCAAGGTGAGCTGCGTCAGTCAAAATGGCCACAGCTTTCGCAGCGTTATCCGTACCT |
| G81I | AACAACGATGACTGGGCGGGTCTCTCCTGCTTCGTTGAGCACCTCAAGGATAGCTGCGTCAGTCAAAATGGCCACAGCTTTCGCAGCGTTATCCGTACCT |
| G81K | AACAACGATGACTGGGCGGGTCTCTCCTGCTTCGTTGAGCACCTCAAGCTTAGCTGCGTCAGTCAAAATGGCCACAGCTTTCGCAGCGTTATCCGTACCT |
| G81L | AACAACGATGACTGGGCGGGTCTCTCCTGCTTCGTTGAGCACCTCAAGCAGAGCTGCGTCAGTCAAAATGGCCACAGCTTTCGCAGCGTTATCCGTACCT |
| G81M | AACAACGATGACTGGGCGGGTCTCTCCTGCTTCGTTGAGCACCTCAAGCATAGCTGCGTCAGTCAAAATGGCCACAGCTTTCGCAGCGTTATCCGTACCT |
| G81N | AACAACGATGACTGGGCGGGTCTCTCCTGCTTCGTTGAGCACCTCAAGGTTAGCTGCGTCAGTCAAAATGGCCACAGCTTTCGCAGCGTTATCCGTACCT |
| G81P | AACAACGATGACTGGGCGGGTCTCTCCTGCTTCGTTGAGCACCTCAAGTGGAGCTGCGTCAGTCAAAATGGCCACAGCTTTCGCAGCGTTATCCGTACCT |
| G81Q | AACAACGATGACTGGGCGGGTCTCTCCTGCTTCGTTGAGCACCTCAAGCTGAGCTGCGTCAGTCAAAATGGCCACAGCTTTCGCAGCGTTATCCGTACCT |
| G81R | AACAACGATGACTGGGCGGGTCTCTCCTGCTTCGTTGAGCACCTCAAGGCGAGCTGCGTCAGTCAAAATGGCCACAGCTTTCGCAGCGTTATCCGTACCT |
| G81S | AACAACGATGACTGGGCGGGTCTCTCCTGCTTCGTTGAGCACCTCAAGGGAAGCTGCGTCAGTCAAAATGGCCACAGCTTTCGCAGCGTTATCCGTACCT |
| G81T | AACAACGATGACTGGGCGGGTCTCTCCTGCTTCGTTGAGCACCTCAAGGGTAGCTGCGTCAGTCAAAATGGCCACAGCTTTCGCAGCGTTATCCGTACCT |
| G81V | AACAACGATGACTGGGCGGGTCTCTCCTGCTTCGTTGAGCACCTCAAGCACAGCGGCGTCAGTCAAAATGGCCACAGCTTTCGCAGCGTTATCCGTACCT |
| G81W | AACAACGATGACTGGGCGGGTCTCTCCTGCTTCGTTGAGCACCTCAAGCCAAGCTGCGTCAGTCAAAATGGCCACAGCTTTCGCAGCGTTATCCGTACCT |
| G81Y | AACAACGATGACTGGGCGGGTCTCTCCTGCTTCGTTGAGCACCTCAAGGTAAGCTGCGTCAGTCAAAATGGCCACAGCTTTCGCAGCGTTATCCGTACCT |

**Table S3:** Generated strains carrying a *murE*-G81 mutation and their specific L-lysine production and fluorescence

| **Codon**  **241-243** | **Amino acid 81** | **Fluorescence (AU)** | **L-Lysine**  **(mM)** | **Codon**  **241-243** | **Amino acid 81** | **Fluorescence (AU)** | **L-Lysine**  **(mM)** |
| --- | --- | --- | --- | --- | --- | --- | --- |
| TGC | Cysteine (C) | 0.16 | 0.00 | CGC | Arginine (R) | 2.05 | 12.26 |
|  |  | 1.08 | 7.29 |  |  | 2.23 | 12.85 |
|  |  | 1.08 | 7.40 |  |  | 2.05 | 9.55 |
|  |  | 1.12 | 8.77 | TCC | Serine (S) | 0.18 | 0.00 |
|  |  | 1.13 | 7.68 |  |  | 0.19 | 0.00 |
|  |  | 1.22 | 4.59 |  |  | 0.20 | 0.00 |
| TTC | Phenylalanine (P) | 1.36 | 8.19 |  |  | 0.20 | 0.04 |
|  |  | 1.21 | 9.39 | TGG | Threonine (T) | 0.47 | 1.46 |
|  |  | 1.36 | 9.52 |  |  | 1.12 | 5.92 |
|  |  | 1.41 | 8.46 |  |  | 1.11 | 7.70 |
| CAC | Histidine (H) | 0.46 | 0.63 | GTG | Valine (V) | 1.47 | 9.49 |
|  |  | 0.72 | 3.50 |  |  | 1.49 | 9.12 |
| ATC | Isoleucine (I) | 1.85 | 10.18 |  |  | 1.56 | 8.00 |
| CTG | Leucine (L) | 1.80 | 12.03 | TGG | Tryptophan (W) | 2.39 | 17.16 |
|  |  | 1.83 | 12.06 |  |  | 1.68 | 9.15 |
|  |  | 1.92 | 12.09 |  |  | 1.80 | 11.30 |
|  |  | 1.97 | 11.76 | TAC | Tyrosine (Y) | 1.10 | 7.10 |
|  |  | 1.93 | 12.23 |  |  | 1.11 | 9.22 |
| AAC | Asparagine (N) | 0.67 | 4.17 |  |  | 1.10 | 9.13 |
|  |  | 0.73 | 4.62 |  |  | 1.15 | 6.77 |
|  |  | 0.48 | 1.29 |  |  | 0.45 | 0.87 |
|  |  | 0.71 | 4.35 |  |  | 1.17 | 8.86 |

KanR (-) TGG CTG ACG GAA TT**C** TAT GCC TCT T 3’

ACC GAC TGC CTT AA**G** ATA CGG AGA A 5’

**Oligo 5’-3’** **Sequence KanR cfu**

Kan100 TGG CTG ACG GAA TTT ATG CCT CTT 2.7x10^6^

Kan100-a48g --- --- --- --G --- --- --- --- 7.5x10^4^
Kan100-g45c --- --- --C --- --- --- --- --- 2.7x10^6^

Kan100-g42c --- --C --- --- --- --- --- --- 3.7x10^6^

Kan100-t51c --- --- --- --- --C --- --- --- 4.0x10^6^

Kan100-t57c --- --- --- --- --- --- --C --- 3.5x10^6^

Kan100-all --- --C --C --G --C --- --C --- 9.2x10^5^

**Figure S1:** Effect of multiple changes on oligo recombination frequencies. The double-strand sequence of the region around the KanR mutation leading to a frameshift (yellow) is shown at the top. All oligos are 60 bases in length, and only relevant changes are shown. Recombination values obtained from these oligos are normalized per 10^9^ viable cells.
